# 

**Published:** 1854-02

**Authors:** 


### CONTENTS OF FEBRUARY NUMBER.

| | Page | | Page |
|---|---|---|---|
| Invalids and Exertive Exercise | 25 | Horseback Exercise | 40 |
| Common Sense | 27 | Going to the South | 41 |
| Longevity | 30 | Would a glass of Wine hurt me? | 42 |
| Ventilation | 31 | How to Sleep | 43 |
| Ignorance, fatal effects of | 33 | To Correspondents | 47 |
| Over-working | 33 | A Premium | 48 |
| Air and Exercise | 35 | | |

